# Improving mental health outcomes of Chinese women survivors of intimate partner violence through advocacy interventions

**DOI:** 10.1017/gmh.2018.5

**Published:** 2018-04-17

**Authors:** Agnes Tiwari, Denise Shuk Ting Cheung, Vivian Hui

**Affiliations:** 1 School of Nursing, Hong Kong Sanatorium & Hospital, Hong Kong; 2 School of Nursing, The University of Hong Kong, Hong Kong; 3 St. Teresa Hospital, Hong Kong

**Keywords:** Advocacy intervention, mental health, intimate partner violence, Chinese women

## Introduction

Intimate partner violence (IPV), one of the two most common forms of violence against women, is a prominent public health problem worldwide (https://www.cdc.gov/violenceprevention/intimatepartnerviolence/definitions.html). For example, the World Report on Violence and Health revealed that between 10% and 69% of women reported being physically assaulted by an intimate male partner at some point in their lives. (http://www.who.int/violence_injury_prevention/violence/global_campaign/en/chap4.pdf). International research has repeatedly shown that violence against women by their intimate male partners is widespread and an important determinant of health for women and children (Heise & Garcia-Moreno, 2002; Garcia-Moreno *et al.*, 2006). Not only are the physical health consequences of IPV well documented (Campbell, 2002), an increased risk of mental health disorders has also been shown to be associated with IPV victimization (Oram *et al.*, 2016).

While different forms of interventions exist for survivors of IPV (e.g. psychological, pharmacological, and advocacy), intervention effectiveness is equivocal across studies (Vickerman & Margolin, 2009; Bisson *et al.*, 2013; Rivas et al., 2015). Further, only a few studies have reported on the interventions’ beneficial impact on survivors’ mental health (Kiely *et al.*, 2010; Jahanfar *et al.*, 2014). More work is also needed to ascertain if the interventions are effective in addressing the needs of abused women in diverse and marginalized groups (NICE, 2014). Likewise, the need to tailor interventions for cultural variations among women survivors of IPV was also highlighted in a Cochrane review of 13 trials involving 2141 abused women from various ethnic groups (Rivas *et al.*, 2015).

This paper will present four advocacy interventions specially designed for abused Chinese women in Hong Kong. As the women are from diverse groups of the Chinese population, the interventions are therefore tailored for the specific groups. Of the four interventions, one is for abused pregnant Chinese women, another one targets abused Chinese women in shelters, yet another is for community-dwelling abused Chinese women, and there is also an intervention for abused immigrant Chinese women. In addition, the interventions also take into account the cultural variations among the women. For example, immigrant women from China are likely to have cultural norms and practices that differ from those found among women born and bred in Hong Kong. Even among local Chinese women, pregnant women, being of a younger age group, are likely to have different values and beliefs compared with the older community-dwelling women.

We shall explain how we seek to meet the specific needs of the women and highlight the mental health outcomes of the interventions. The lessons that we have learned from our experiences of using advocacy interventions in a Chinese cultural context will also be expounded.

## Advocacy interventions for Chinese women survivors of IPV

Advocacy interventions are based on the concept of empowerment (Dutton, 1992). In practice, an advocate (e.g. a health professional) empowers an abused woman by helping her understand the abuse and her response to it. Instead of telling the woman what to do, the advocate talks through potential solutions with her and assists her to set goals and work towards them. Safety planning, provision of legal, housing and financial advice, facilitating access to and use of community resources, and ongoing support and informal counseling are key components of advocacy interventions (Rivas *et al.*, 2015). Four advocacy interventions specially designed for abused Chinese women are presented as follows.

### A modified empowerment intervention for abused pregnant Chinese women

The intervention is based on an empowerment protocol developed by Parker *et al.* (1999). Intended to enhance abused women's independence and control, the original protocol focused on safety planning, choice making, and problem-solving. When the intervention was first adopted for use in a group of abused pregnant Chinese women (Tiwari *et al.*, 2005), modifications were undertaken to ensure cultural congruence. For example, we suggested to the women that they considered including ‘trusted friends and neighbors’ (instead of ‘friends and neighbors’ as in the original protocol) in their safety planning. The reason is that domestic violence is often viewed as a shame and Chinese women may be very reluctant to disclose it to someone outside the family. We also added empathic understanding to the protocol with an emphasis on taking in and accepting women's feelings and perceptions about the abuse. Such an addition was necessary because the majority of the abuse reported was psychological. In the absence of wounds and injuries, the women's experience of abuse was often played down or even ignored by others. The 30-min intervention, which was administered once, one-on-one, by a trained research nurse in the antenatal period, was shown to be effective in lowering postnatal depression scores (Tiwari *et al.*, 2005). Specifically, the women found much relief when they were listened to without the fear of being judged by others.

### An intensive empowerment intervention for Chinese women in shelter

The 6-h intervention, delivered over a 3-week period, was designed to meet the physical, psychological, social and legal needs of abused Chinese women with a history of severe and/or repeated IPV victimization while they were taking refuge in a shelter (Tiwari *et al.*, 2010b). The intervention consisted of (i) the modified empowerment intervention as described above, (ii) parenting skills and management of children's behavioral problems, (iii) group counseling and legal advocacy, and (iv) health assessment and dietary teaching based on the concepts of Chinese Medicine. We intended that the teaching of parenting skills and management of children's behavioral problems would enhance the women's confidence in their own parenting ability. Group counseling and legal advocacy were designed to improve their understanding of and responding to the social construction of women as legitimate victims of partner violence in Chinese society. Health teaching based on the concept of Chinese Medicine aimed to help them make health choices and achieve optimal health outcomes. In a randomized controlled trial (Tiwari *et al.*, 2010b), the effect of the intervention on the women's mental health was evaluated. The results were somewhat mixed. Although the women reported significant improvement in depressive symptoms at 6-month follow-up, the improvement was not significantly different from that reported by women receiving standard care. Women who adhered to the Chinese dietary regimen, however, reported significant improvement in physical symptoms. Parenting and decision-making skills were also reported to have improved significantly (Tiwari *et al.*, 2010b).

### An advocacy intervention for community-dwelling abused Chinese women

Over a 12-week period, abused Chinese women recruited from a community in Hong Kong received an advocacy intervention consisting of (i) a one-off, 30-min modified empowerment intervention with empathic understanding, and (ii) 12 scheduled weekly telephone calls for social support by a trained social worker (Tiwari *et al.*, 2010a). Empowerment intervention was chosen for the same reason as that for abused pregnant women (Tiwari *et al.*, 2005) (psychological abuse in the absence of physical abuse and fear of not being taken seriously deterred the women in reporting abuse and/or seeking help). The use of telephone calls to provide formal social support was based on the belief that abused women are generally resourceful and, with support from professionals whom they can trust, they are capable of coping with the challenges of IPV victimization. The intervention was evaluated in a randomized controlled trial (Tiwari *et al.*, 2010a). The participants reported a significant reduction in depressive symptoms after the intervention as measured by the Chinese Beck Depression Inventory-II (BDI-II) when compared with those who received standard community care. However, as it was less than a 5-unit reduction in BDI-II scores, the mental health improvement was not considered as clinically important (Furukawa, 2010). In a subsequent secondary analysis of the same database, an unexpectedly high proportion of immigrant women were found among the study participants. Further, immigrant status, social isolation and parenting stress were clearly identified as risk factors for experiencing depressive symptoms in this group of community-dwelling abused women (Wong *et al.*, 2011). The findings pointed to the need for identifying and catering for the special needs of abused immigrant women when designing advocacy interventions.

### A purpose-built empowerment intervention for abused immigrant Chinese women

Based on the lessons learned, we designed an empowerment intervention for abused immigrant women from China. In the 12-week intervention, we retained the one-off empowerment with empathic understanding and the 12 weekly telephone calls for social support while adding two more components. A Child Friendly Parenting component, based on the UNICEF Child Friendly City Framework, was added to enable the women, conduct parenting free from the use of physical force. As the use of physical punishment in parenting was forbidden in Hong Kong but not in China, the discrepancy was often a source of conflict for immigrant parents in Hong Kong. Another added component was peer support provided by trained peers, many of whom were immigrants with experiences of successfully adapting to a new host country and overcoming social isolation. The intervention was shown to be effective in alleviating negative mental health problems including depressive symptoms in a randomized controlled trial (Tiwari *et al.*, 2015).

## Lessons learned

During the course of designing and modifying advocacy interventions for Chinese women survivors of IPV, we have learned some valuable lessons.

- Similar to what has been shown by abused women in non-Chinese cultures (http://apps.who.int/iris/bitstream/10665/77432/1/WHO_RHR_12.36_eng.pdf), abused Chinese women are not passive victims. Rather, they are resilient and resourceful in the face of adversities. This may explain why they respond positively to advocacy interventions.
- It is essential to assess the cultural beliefs of both survivors and perpetrators of IPV when designing an intervention as some traditions (e.g. Confucian tradition) may condone violence against women by an intimate partner as a legitimate treatment and moderate the effect of the intervention.
- While ‘face-saving’ is an important consideration for Chinese women when deciding whether to acknowledge or seek help for the abuse, they would respond more favorably to offers of help from those whom they trust.
- Cultural beliefs are complex and may evolve with time and social/political changes. It is only partially correct to say that Chinese women are victims of IPV due to their submissive and subordinate positions. Since the Communist Revolution in 1949, women's rights in China have greatly improved with men and women treated as equals under the Community Party ideology. Yet, such liberation has not protected women from violence and abuse. Indeed, some have suggested that violence against women may even increase as they gain more power (Koenig *et al.*, 2003). Thus, while it is important to empower abused Chinese women in recognizing their rights and making choices, it is equally important to help them develop negotiation skills as part of safety planning and problem-solving.
- We have learned that Chinese women in our intervention studies do not treat their body and mind as separate entities, which is consistent with what is suggested in the literature (Yick & Agbayani-Siewert, 1993). Also, a previous study of Japanese abused women found that a physical symptom (being physically ill) was a culturally acceptable way of coping with anxiety and depression (Weingourt *et al.*, 2001). It is possible that abused Chinese women may also express their mental health problems through physical symptoms. Therefore, when evaluating the effect of advocacy intervention on Chinese women's mental health, one should be sensitive to their reports of somatic problems which may be a psychosomatic response to the abuse.
- During the course of working on advocacy interventions for abused Chinese women, we have not found a single model that fits all. Instead, through rigorous testing and model refinement, we are closer to finding models of interventions that better match the myriads of needs across the diverse groups of abused women in Chinese society.
- Finally, we have found that while theoretical frameworks and empirical evidence provide robust bases for building interventions, evaluation of their appropriateness for the target group must be conducted so that modifications can be made to ensure a good fit between the intervention model and the recipients.

## Conclusion

The four advocacy interventions presented in this paper underscore the need to adopt culturally appropriate models and the importance of incorporating theoretical frameworks and empirical evidence when constructing models of intervention for survivors of IPV victimization.
